# Nuclei Isolation From Murine and Human Periosteum For Transcriptomic Analyses

**DOI:** 10.21769/BioProtoc.5211

**Published:** 2025-02-20

**Authors:** Simon Perrin, Cassandre Goachet, Maria Ethel, Yasmine Hachemi, Céline Colnot

**Affiliations:** Univ Paris Est Creteil, INSERM, IMRB, Creteil, France

**Keywords:** Nuclei isolation, Single-nucleus RNA-seq, Periosteum, Fracture callus, Fluorescence-activated nuclei sorting

## Abstract

Bone repair is a complex regenerative process relying on skeletal stem/progenitor cells (SSPCs) recruited predominantly from the periosteum. Activation and differentiation of periosteal SSPCs occur in a heterogeneous environment, raising the need for single cell/nucleus transcriptomics to decipher the response of the periosteum to injury. Enzymatic cell dissociation can induce a stress response affecting the transcriptome and lead to overrepresentation of certain cell types (i.e., immune and endothelial cells) and low coverage of other cell types of interest. To counteract these limitations, we optimized a protocol to isolate nuclei directly from the intact periosteum and from the fracture callus to perform single-nucleus RNA sequencing. This protocol is adapted for fresh murine periosteum, fracture callus, and frozen human periosteum. Nuclei are isolated using mechanical extraction combined with fluorescence-based nuclei sorting to obtain high-quality nucleus suspensions. This protocol allows the capture of the full diversity of cell types in the periosteum and fracture environment to better reflect the in vivo tissue composition.

Key features

• Allows the isolation of nuclei with high-quality RNA for transcriptomic analyses.

• Can be adapted to be used on fresh and frozen tissue.

• Optimized for human and murine periosteum.

## Graphical overview


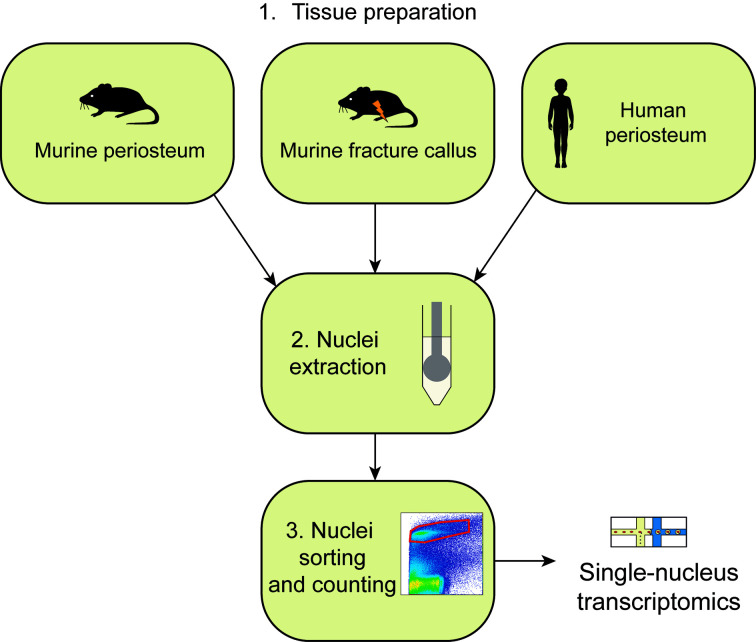


## Background

The periosteum is a thin and heterogeneous tissue covering the outer surface of bone that contains skeletal stem/progenitor cells (SSPCs) essential for bone repair. Following a bone fracture, periosteal SSPCs are activated and differentiate to form cartilage and bone cells [1–4]. SSPCs differentiate in a highly complex and dynamic environment marked by the presence of many cell types, including immune cells, endothelial cells, and cells from the nervous system [5,6]. To decipher the heterogeneity of the periosteum and the response of these different cell types to bone fracture, we aimed to perform single nucleus/cell transcriptomics from the periosteum and callus tissues. Cell isolation using enzymatic digestion is commonly used, but there are several disadvantages to this method. First, enzymatic tissue digestion can favor the representation of certain cell types. This usually leads to the overrepresentation of cell types loosely attached to the matrix, such as immune cells, and to the limited coverage of cell types embedded in the matrix, including osteoblasts, osteoclasts, and Schwann cells. Second, FACS-sorting and single cell transcriptomic techniques have constraints in terms of the cell size that can be processed. The fracture callus contains large cells such as hypertrophic chondrocytes and osteoclasts that are not compatible with these techniques. Third, enzymatic digestion needed for single-cell RNA sequencing (scRNAseq) can only be performed on fresh tissues and induces a stress response leading to transcriptomic changes[8]. To overcome these limitations, we optimized and tested a protocol to isolate nuclei from fresh and frozen tissues to perform single-nucleus RNA sequencing (snRNAseq). Several studies have shown that snRNAseq can produce similar results as scRNAseq and capture a greater diversity of cell types [7–11]. Here, we detail three distinct ways of using our protocol in order to isolate nuclei from (i) fresh murine periosteum, (ii) fresh murine fracture hematoma/callus, and (iii) frozen human periosteum. While tissue preparation can change between the different types of samples, the nuclei extraction protocol is common and based on mechanical cell lysis. Nuclei extraction is followed by fluorescence-activated nuclei sorting to eliminate the yields of cellular and matrix debris in the suspension. Overall, this protocol is fast and easy-to-use and is currently the most adequate method to capture the periosteal heterogeneity in vivo.

## Materials and reagents

**Biological materials**

1. 8–12-week-old mice, C57BL6 background (Janvier Labs, France, or equivalent vendors). We used a mix of males and females in our experiments

2. Periosteum from patients. We obtained fresh samples from patients undergoing surgery and processed them up to 4 h after tissue collection. Sample collection from patients requires approval by the Ethics Committee and formal consent from the donor

**Reagents**

1. DMEM medium (Life Technologies, catalog number: 11966025)

2. HEPES buffer (Thermo Fisher, catalog number: 15630056)

3. Penicillin/streptomycin (pen/strep) (Life Technologies, catalog number: 15140122)

4. PBS, RNase free (Thermo Fisher, catalog number: AM9624)

5. Ethanol absolute (VWR, catalog number: 20821.365)

6. Nuclei lysis buffer (Sigma-Aldrich, catalog number: NUC101-1KT)

7. Bovine serum albumin, molecular biology grade (Merck, catalog number: B6917)

8. RNase inhibitor (Roche, catalog number: 3335399001)

9. DNase/RNase-free distilled water, UltraPure (Life Technologies, catalog number: 10977049)

10. SYTOX^TM^ AADvanced^TM^ Dead Cell Stain kit (Thermo Fisher, catalog number: S10349)

11. DAPI (Life Technologies, catalog number: D3571), resuspend in distilled water at a concentration of 5 mg/mL

12. Buprenorphine (Centravet, catalog number: BUP001)

13. Atipamezole (Centravet, catalog number: ANT201)

14. Ketamine (Centravet, catalog number: KET205)

15. Medetomidine (Centravet, catalog number: DOM003)

**Solutions**

1. Human sample collection medium (see Recipes)

2. 70% ethanol (see Recipes)

3. Nuclei suspension buffer (see Recipes)

4. DAPI buffer (see Recipes)

**Recipes**

**1. Human sample collection medium**

Solution can be stored for up to 1 year at 4 °C.

**Table:** 

Reagent	Final concentration	Volume
DMEM	1×	44.5 mL
HEPES	10%	5 mL
Pen/strep	1%	0.5 mL
Total	n/a	50 mL

**2. 70% ethanol**

**Table:** 

Reagent	Final concentration	Volume
Ethanol (absolute)	70%	700 mL
H_2_O	n/a	300 mL
Total	n/a	1,000 mL

**3. Nuclei suspension buffer**

Make fresh for each experiment and keep at 4 °C on ice.

**Table:** 

Reagent	Final concentration	Amount
RNase-free PBS (10×)	1×	0.5 mL
Bovine serum albumin	2%	0.1 g
RNase inhibitor (40 U/µL)	0.2 U/µL	25 µL
RNase-free water	n/a	4.5 mL
Total	n/a	5 mL

**4. DAPI buffer**

Make fresh for each experiment and protect from the light.

**Table:** 

Reagent	Final concentration	Volume
Nuclei suspension buffer	1×	99 µL
DAPI	1/1,000	1 µL
Total	n/a	100 µL

**Laboratory supplies**

1. Conical tubes, 15 and 50 mL (Falcon, catalog numbers: 352097 and 352070 or equivalent)

2. Eppendorf tubes 1.5 and 0.2 mL

3. Cryotube ClearLine^®^ 2 mL (Dutscher, catalog number: 390701)

4. Pipettes 10 and 25 mL (Dutscher, catalog numbers: 357551 and 357535 or equivalent)

5. Pipette tips 1 mL, 200 µL, 20 µL, and 10 µL

6. Falcon 5 mL round-bottom polystyrene tubes (Corning, catalog number: 352235)

7. Sterilin^TM^ Quickstart universal containers, PS, 30 mL (VWR, catalog number: 128AR/IRR, or equivalent)

8. Sterile scalpels (Dutscher, catalog number: 132622)

9. Cell strained 40 µm and 100 µm (Fisher Scientific, catalog numbers: 352340 and 352360)

10. 25 G needles (Terumo, catalog number: AN*2516R1)

11. 1 mL syringes (Terumo, catalog number: SS+01H1)

12. Greiner Bio-One Petri dishes (bacterial dish) (Dutscher, catalog number: 633185)

13. Kova^®^ slides (Fisher Scientific, catalog number: 22-270141)

14. Liquid nitrogen

15. Ice

## Equipment

1. Centrifuge with temperature control for Falcon 50 mL tubes

2. Centrifuge with temperature control for Eppendorf 1.5 mL tubes

3. BD Influx Cell Sorter or equivalent

4. Zeiss Imager D1 AX10 light microscope (Carl Zeiss Microscopy), or equivalent

5. -80 °C freezer

6. Sterile hood

7. Microvolume pipettes

8. Surgical forceps (Dumont AA Forceps) (FST, catalog number: 11210-20 or equivalent)

9. Surgical scissors (Fine Scissors-ToughCut^®^ 11 mm) (FST, catalog number: 14058-11 or equivalent)

10. Dissecting chisel (Fine Science Tools, catalog number: 10095-12)

11. Drill (Dremel, catalog number: 8050-15)

12. Drill bits (0.4 mm)

13. Heating pad (Harvard Apparatus, catalog number: 55-7033)

14. Trimmer (Kerbl, catalog number: GT416)

15. 15 mL Dounce homogenizer with pestle A (loose) and pestle B (tight) (Sigma-Aldrich, catalog number: D9938)

16. Liquid nitrogen container

17. Ice container

## Procedure

**A. Prepare material and reagents**

1. Set the centrifuge to 4 °C and allow it to cool before use.

2. Prepare all solutions needed for the protocol.

*Note: Prepare all reagents in RNase-free conditions. All solutions should be prepared fresh and kept on ice.*

*Note: The protocol includes two solutions for nuclei processing. The first solution is the nuclei lysis buffer, used to induce cell lysis. The second solution is the nuclei suspension buffer (see Recipes) and corresponds to the buffer used for centrifugation, sorting, and counting of nuclei.*

3. Prepare and annotate all tubes needed for the procedure.

*Note: To improve the quality of the RNA preparation, it is recommended to work in RNase-free conditions, by using RNase-free solutions and RNase-free materials (tubes, dishes, dissection tools, douncer), and working on clean RNase-free surfaces (use appropriate reagent to clean benches).*

**B. Option I: Nuclei isolation from fresh murine periosteum (Timing: up to 30 min)**

1. Sacrifice the mice by cervical dislocation (or any other appropriate method).

*Note: As the periosteum is very thin in adult mice, we recommend using the tibias of at least five mice.*

*Note: All procedures involving animals must be approved by ethical committees.*

**Figure 1.**
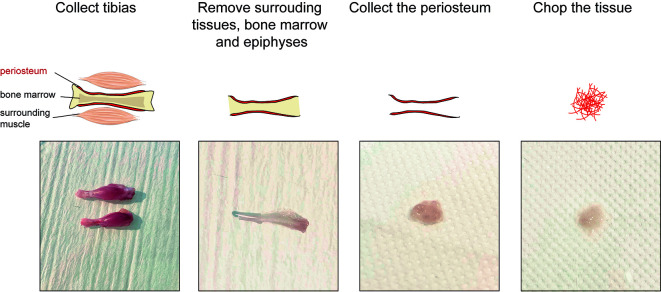
Tissue processing of uninjured murine periosteum

2. Rinse the limbs with 70% ethanol.

3. Incise the skin and remove it entirely from the lower limbs. Disconnect the tibias from the limbs by cutting at the knee and ankle level. Place the tibias in a 100 mm sterile Petri dish with RNase-free chilled PBS on ice (Figure 1, left).

4. Remove the soft tissues surrounding the tibias using forceps and scissors (Figure 1, middle left).

*Note: Remove soft tissue gently using scissors, as pulling out muscle could detach the periosteum from the cortex.*

*Note: Dissection steps can be performed under a binocular microscope to reduce the risk of contamination by surrounding tissue.*

5. Cut the epiphyses of the tibia using scissors. Flush the bone marrow using RNase-free ice-cold PBS.

6. Collect the periosteum by scraping it from the cortex using a dissecting chisel. Collect the tissue in a 1.5 mL Eppendorf containing ice-cold lysis buffer and place it on ice (Figure 1, middle right).

7. Put the tissues in a 100 mm sterile Petri dish with a drop of ice-cold lysis buffer. Chop it with clean scissors until very small pieces are obtained (Figure 1, right).

8. Proceed immediately to nuclei extraction (section E).

**Critical:** Do not wait to perform nuclei extraction; any time lost will impact RNA quality.

**C. Option II: Isolation of fresh murine fracture hematoma/callus (Timing: up to 30 min)**

1. Induce tibial fracture.

*Note: All procedures involving animals must be approved by ethical committees.*

*Note: We recommend using at least five mice for post-fracture day 1 and at least three mice for later time points to obtain a sufficient number of nuclei and overcome interindividual variability.*

a. Anesthetize the mice with an intraperitoneal injection of 50 mg/kg ketamine and 1 mg/kg medetomidine. Inject 0.1 mg/kg of buprenorphine subcutaneously for analgesia.

b. After 15–30 min, if the quality of the anesthesia and analgesia is sufficient, shave the right limb and sanitize using skin disinfectant.

*Note: The efficiency of anesthesia must be checked using foot pinching or other appropriate techniques.*

**Figure 2.**
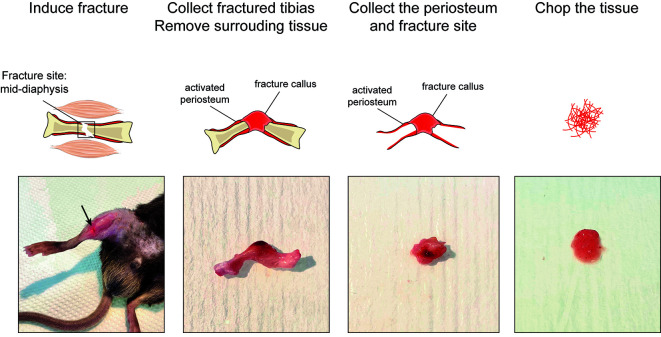
Tissue processing of murine fracture callus and activated periosteum

c. Perform a 2 cm incision on the skin along the tibia using a sterile scalpel and expose the tibial surface by gently separating the muscles from the bone surface.

d. Create three holes in the mid-diaphysis aligned perpendicular to the tibial axis using a drill and a 0.4 mm drill bit.

e. Induce osteotomy by cutting the bone along the three holes with scissors (Figure 2, left).

f. Close the skin wound using suture threads.

g. Revive the mice with an intraperitoneal injection of 1 mg/kg atipamezole and place it on a 37 °C heating pad until revived.

h. Perform two additional subcutaneous injections of buprenorphine 0.1 mg/kg at 12 and 24 h post-surgery and monitor the mice closely until callus tissue collection.

2. Collect tissue for nuclei extraction.

*Note: We tested our protocol on fracture tissue up to 7 days post-fracture. Later time points may need further optimization as the fracture callus becomes more ossified.*

a. Sacrifice the mice by cervical dislocation (or any other appropriate method) and rinse the limbs with 70% ethanol.

b. Incise the skin and remove it entirely from the fractured limb. Disconnect the tibia from the limb by cutting at the knee and ankle level and place it in a 100 mm sterile Petri dish with RNase-free chilled PBS.

*Note: Proceed very gently to avoid separating the two segments of the tibia while dissecting, as the fractured tibia is very fragile in the first days post-injury.*

3. Remove the soft tissues surrounding the tibia using forceps and scissors (Figure 2, middle left).

*Note: Remove the soft tissue gently using scissors, as pulling out muscle could damage the fracture tissue.*

4. Scrape the fracture hematoma/callus and the activated periosteum from the diaphysis using a dissecting chisel. Collect the tissue in ice-cold lysis buffer placed on ice (Figure 2, middle right).

5. Put the tissue with a drop of ice-cold lysis buffer. Chop it with clean scissors until very small pieces are obtained (Figure 2, right).

6. Proceed immediately to nuclei extraction (section E).

**Critical:** Do not wait to perform nuclei extraction; any time lost will impact RNA quality.

**D. Option III: Isolation of frozen human periosteum**

**Figure 3.**
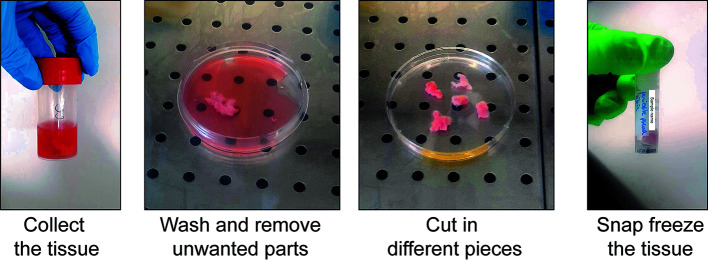
Collection and freezing of the human periosteum

1. Collect and freeze the human periosteum.

a. Place the sample in a tube containing at least 10 mL of ice-cold human sample collection medium immediately after collection. Place at 4 °C until processing (Figure 3, left).

*Note: Samples should be processed as early as possible after collection. In our experience, samples can be processed up to 3 h after collection and used for nuclei extraction.*

b. Under a cell culture hood, remove the tissue from the tube and place it in a sterile Petri dish. Wash the tissue with RNase-free ice-cold PBS to remove the remaining medium and blood (Figure 3, middle left).

c. Using scissors, scalpel, and tweezers, remove any muscle, fat tissue, or damaged tissue.

d. Cut the periosteum into pieces of at least 0.5 × 0.5 cm. Place the pieces briefly in a dry Petri dish to remove the medium from the tissue (Figure 3, middle right).

*Note: We recommend storing larger pieces of tissue to obtain a higher number of nuclei after nuclei extraction. Store at least three pieces of tissues if possible.*

e. Place each piece of periosteum in a properly labeled freezing tube.

f. Place the tubes directly in liquid nitrogen and leave for at least 5 min. Then, place the tubes at -80 °C (Figure 3, right).

**Caution:** Handle liquid nitrogen with care and follow safety rules.

**Stop point:** Samples can be stored at -80 °C for a long time before processing. We have used samples for up to three years after freezing.

2. Check RNA integrity.

a. If possible, use one sample from the same batch to extract RNA and check that RNA integrity is above 6.5. We routinely used the RNeasy Mini Kit following the manufacturer’s instructions for RNA extraction followed by RIN measure on Agilent TapeStation, but any equivalent methods can be used.

*Note: We avoid using samples with RIN lower than 6.5 as it may lead to poor RNA quality after nuclei extraction.*

**Figure 4.**
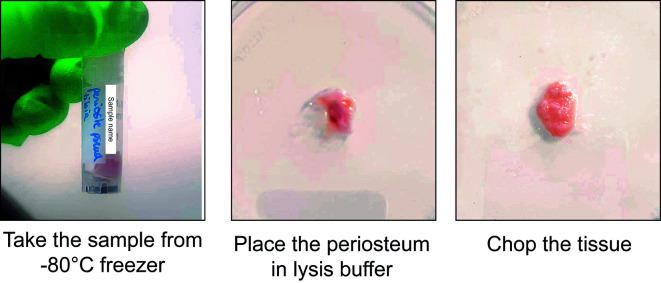
Tissue processing of human periosteum

3. Prepare tissue for nuclei extraction (Timing: up to 15 min).

a. Take the sample from the -80 °C freezer and put it on dry ice (Figure 4, left).

**Critical point:** Keep the tube on dry ice until it is used for extraction. Do not allow the sample to thaw before placing it into the lysis buffer.

b. Put the frozen tissue on an RNase-free Petri dish placed on ice. Add a few drops of ice-cold lysis buffer (Figure 4, middle).

c. Chop the tissue with clean scissors until very small pieces are obtained (Figure 4, right).

d. Proceed immediately to nuclei extraction (section E).

**Critical:** Do not wait to perform nuclei extraction; any time lost will lead to a decrease in RNA quality.

**E. Nuclei extraction (Timing: up to 15 min)**

**Figure 5.**
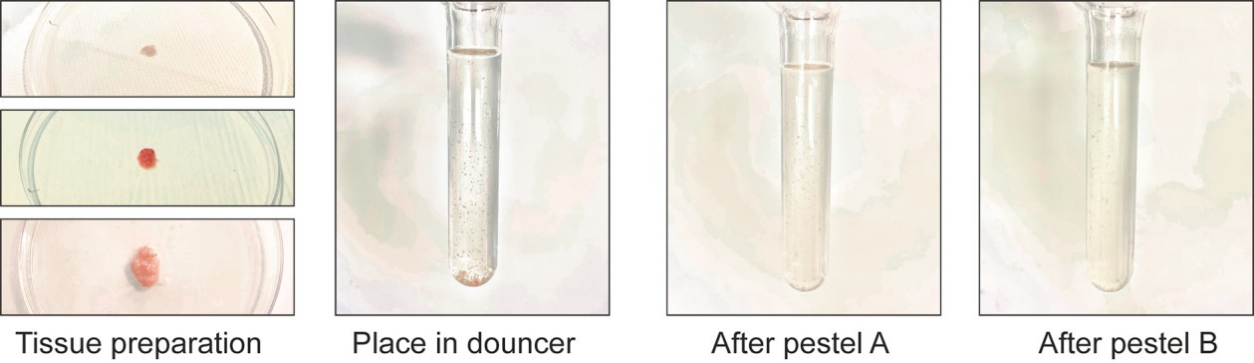
Nuclei extraction from murine and human periosteum

1. Put the tissues prepared in parts B, C, or D in a glass douncer. Add up to 7 mL of ice-cold lysis buffer (Figure 5, left, middle left).

2. Put the douncer on ice for 2 min.

3. While keeping the douncer on ice, lower and raise the pestle A gently 15–20 times, avoiding bubble formation (Figure 5, middle right).

*Note: There shouldn’t be too much resistance while using pestles. Resistance can be due to the presence of big pieces of tissue, which would require better chopping before putting the tissue in the douncer, or to a high ratio of tissue/lysis buffer, which would require putting less tissue or more lysis buffer, if possible.*

4. Lower and raise the pestle B gently up to 10 times (Figure 5, right).

5. Filter the suspension with a 100 µm cell strainer in a 50 mL conical tube. Collect the flowthrough and filter using a 40 µm cell strainer in a clean 50 mL tube.

6. Centrifuge at 500× *g* for 5 min at 4 °C. Carefully remove the supernatant without disturbing the pellet.

7. Add 1 mL of ice-cold nuclei suspension buffer. Resuspend the pellet and transfer to an RNase-free Eppendorf tube.

8. Centrifuge at 500× *g* for 5 min at 4 °C. Carefully remove the supernatant without disturbing the pellet.

9. Resuspend in 200 µL of ice-cold nuclei suspension buffer. Proceed to nuclei sorting.

**F. Nuclei sorting and counting (Timing: up to 30 min)**

1. Nuclei sorting:

a. Add 1 µL of SYTOX^TM^ AADvanced^TM^ in the solution to label DNA in nuclei.

*Note: We also tested our protocol using DAPI staining with similar results.*

**Figure 6.**
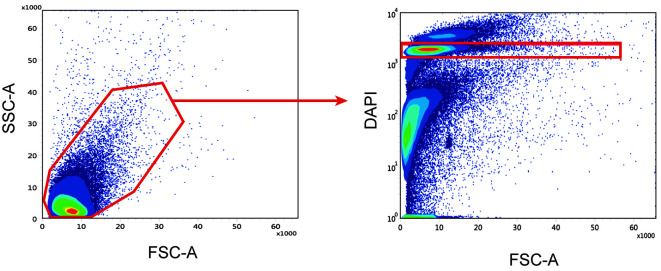
Sorting strategy to obtain a nuclei suspension without debris. The suspension is first gated based on SSC-A and FSC-A to remove larger debris (left), and nuclei are selected based on DAPI fluorescence (right).

b. Sort up to 150,000 nuclei (Sytox AADvanced+ nuclei) using the appropriate gating strategy (Figure 6). Collect in 1.5 mL Eppendorf tubes containing 0.75 mL of ice-cold nuclei suspension buffer.

*Note: Prior to the snRNAseq experiment, cell sorting should be settled with appropriate controls including unstained nuclei.*

c. Centrifuge the suspension of sorted nuclei at 500× *g* for 5 min at 4 °C. Carefully remove the supernatant without disturbing the pellet.

**Critical:** Depending on the number of sorted nuclei, the pellet may not be visible. Proceed carefully to the supernatant removal to avoid eliminating the nuclei.

d. Resuspend in 50 µL of ice-cold nuclei suspension buffer.

2. Nuclei counting (Timing: up to 15 min):

a. Mix 5 µL of the nuclei suspension with 10 µL of DAPI solution in a 0.2 mL tube.

*Note: Adapt the dilution to obtain an optimal number of nuclei for counting. Too much or not enough nuclei during counting could lead to errors.*

**Figure 7.**
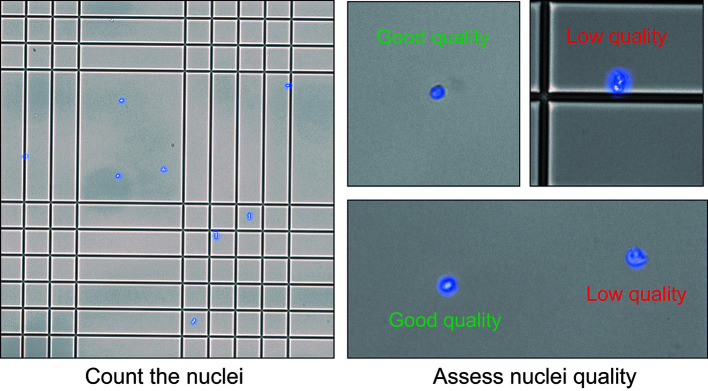
Nuclei counting and quality check. Nuclei should have a clean round shape. Nuclei with abnormal shape, unclear boundaries, and altered membranes are considered low quality.

b. Transfer 10 µL of the stained nucleus suspension to a Kova slide.

c. Under a fluorescence microscope, check the quality of the nuclei suspension. Count the number of nuclei to obtain the concentration of the nucleus suspension (Figure 7).

*Note: We recommend using both brightfield and fluorescence microscopy to check the nuclei suspension.*

**Critical:** It is crucial to assess the quality of the nuclei suspension before proceeding to the next steps of the experiments. The suspension should only contain nuclei and no debris. The shape of the nuclei reflects their quality. If the suspension contains debris or a high percentage of low-quality nuclei, we recommend not to use it for further analysis.

3. Proceed immediately to loading for snRNAseq following the manufacturer’s instructions.

*Note: Using a nuclei solution of 700–1,200 nuclei per microliter is recommended.*

## Validation of protocol

This protocol was adapted from previously published protocols [12,13] and was used in other studies and on various tissues. We have generated more than 10 murine samples and 6 human samples using this protocol, which were published in [3,5,6]. Datasets from the murine uninjured periosteum and fracture callus are available at the following link: https://cells.ucsc.edu/?ds=fracture-repair-atlas.

## General notes and troubleshooting

**Troubleshooting**

Poor data quality after snRNAseq might be due to low-quality nuclei preparation. To improve sample quality, we recommend the following:

1. Work in an RNase-free environment. All solutions used for the protocol must be RNase-free. All tools used for this protocol must be sterile and cleaned to eliminate RNase.

2. Reduce experimental time. Time is crucial to preserve RNA quality. Optimize all steps of nuclei extraction and sorting and process immediately to snRNAseq after nuclei extraction.

3. A poor yield of nuclei can be due to problems in mechanical extraction. The use of the pestle should be smooth. If there is strong resistance while using the pestle, consider increasing the chopping time, reducing the amount of tissue, or increasing the volume of lysis buffer.

4. Ensure that the nuclei counting is accurate and that the suspension does not contain debris.

5. If working with frozen human periosteum, proper snap freezing and cryopreservation are required to obtain high-quality nuclei and RNA. Reduce the time between tissue collection and freezing. To ensure RNA quality, perform RNA extraction and check RNA integrity (RIN) from a sample from the same batch. Samples with a RIN lower than 6.5 will lead to poor results.

6. Differences in age, sex, species, and pathological conditions can vary the yield of recovered nuclei and should be taken into consideration. Adjust the amount of tissue for extraction to compensate.
